# Low abundance of Archaeorhizomycetes among fungi in soil metatranscriptomes

**DOI:** 10.1038/srep38455

**Published:** 2016-12-23

**Authors:** Michal Choma, Jiří Bárta, Hana Šantrůčková, Tim Urich

**Affiliations:** 1 Department of Ecosystem Biology, Faculty of Science, University of South Bohemia, České Budějovice, Czech Republic; 2 Institute of Microbiology, Ernst-Moritz-Arndt University Greifswald, Greifswald, Germany

## Abstract

The Archaeorhizomycetes are recently discovered fungi with poorly resolved ecology. Even their abundance in soil fungal communities is currently disputed. Here we applied a PCR-independent, RNA-based metatranscriptomic approach to determine their abundance among fungi in eleven different soils across Europe. Using small subunit (SSU) ribosomal RNA transcripts as marker, we detected Archaeorhizomycetes in 17 out of 28 soil metatranscriptomes. They had average relative SSU rRNA abundance of 2.0% with a maximum of 9.4% among fungal SSU rRNAs. Network analysis revealed that they co-occur with arbuscular mycorrhizal Glomerales, which is in line with their previously suggested association with plant roots. Moreover, Archaeorhizomycetes ranked among the potential keystone taxa. This metatranscriptomic survey exemplifies the usage of non-targeted molecular approaches for the study of soil fungi. It provides PCR- and DNA-independent evidence for the low abundance of Archaeorhizomycetes in soil fungal communities, although they might be non-negligible players despite their low abundance.

The Archaeorhizomycetes are an enigmatic fungal group that has been until recently without cultured representatives. They represent a deeply-branching class in the Ascomycota and have been detected in many soil ecosystems worldwide, indicating their ubiquity in soils^1^. As their name suggests, they show a strong association with the root environment. However, very limited information is available about their life style and ecology, with direct associations with roots through symbiotic or parasitic interactions and even secondary associations via interactions with other root-associated fungi being discussed^2^.

Several DNA-based PCR studies targeting the fungal internally transcribed spacer (ITS) of ribosomal RNA genes had suggested this group to be ubiquitous and numerically dominant in some soil fungal communities^3^. However, a recent global study of soil fungi could not confirm their high abundance^4^. In response, there arose a discussion about possible PCR primer biases and the true abundance of Archaeorhizomycetes in the latter study and generally in soils^5^,^6^.

The bias in PCR studies brought by primer mismatches and differing amplicon length can be avoided by targeting small subunit ribosomal RNA (SSU rRNA) transcripts instead of genes. This is facilitated by random hexamer-primed reverse transcription in metatranscriptomics approaches^7^. Total RNA extracted from soils is dominated by rRNA fragments (usually more than 90% is rRNA) and thus naturally enriched in target molecules, which enables PCR independent SSU rRNA profiling via direct sequencing of reversely transcribed cDNA. Furthermore, the obtained random-hexamer primed sequence fragments originate from different regions of the SSU rRNA molecule and are therefore insensitive to the presence of introns or PCR primer mismatches. Since ribosomal RNA is quickly degraded, it is a better marker for living cells than DNA used in metagenomics or targeted amplicon analysis^8^. The fact that metatranscriptomics does not suffer from PCR-introduced biases and thus does not discriminate any taxonomic group and on top of that gives information about the composition of the transcriptionally active part of the fungal community distinguishes such approach from standard amplicon sequencing. On the other hand, SSU rRNA profiling of fungi provides not the same high taxonomic resolution as the fungal ITS region used for amplicon sequencing.

This study aimed to assess the abundance of Archaeorhizomycetes in 28 metatranscriptomes^7^,^9^,^10^,^11^,^12^ from 11 soils belonging to different terrestrial biomes across Europe (for details see Table 1). Furthermore, network analysis identified possible fungal interactions partners of Archaeorhizomycetes.

## Results and Discussion

Between 514 and 28640 fungal SSU rRNAs were on average analysed per soil, stemming from metatranscriptomes generated by 454 pyrosequencing and Illumina Hiseq sequencing (see Table 1). Generally, the fungal communities were dominated by Ascomycota (57.6% of all fungal rRNA transcripts), followed by Basidiomycota (23.2%), Glomeromycota (6.4%), Chytridiomycota (3.9%), Mortierellomycotina (2.5%) and Mucoromycotina (0.6%).

**Figure 1:**
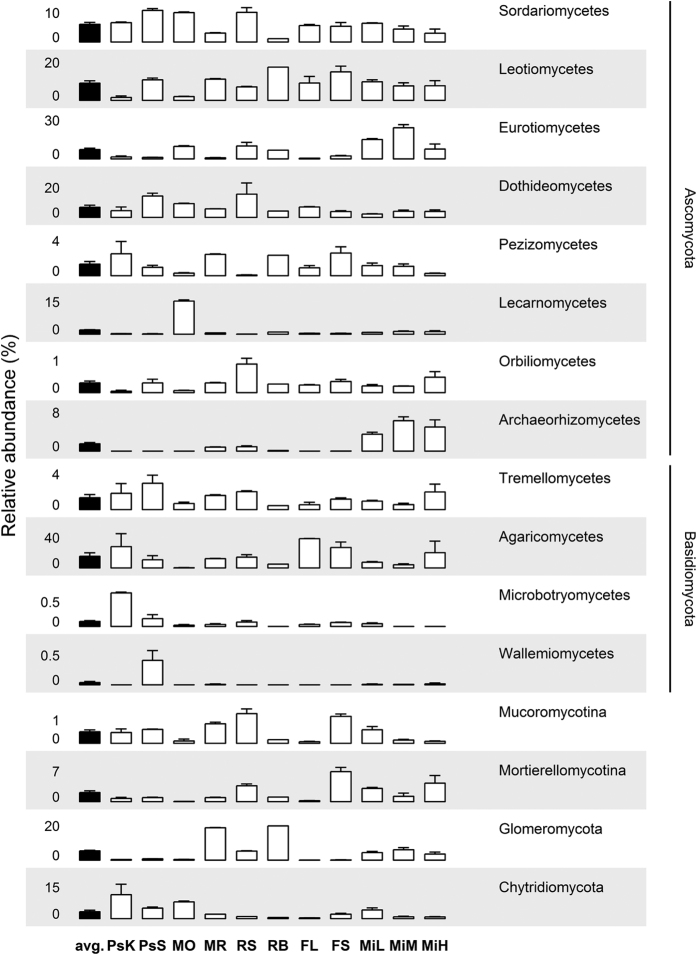
Relative abundance of the 16 most abundant fungal classes (mean + SE) as average of all datasets (black columns) and in respective soils (open columns).

Archaeorhizomycetes were not among the abundant fungal classes (Fig. 1). The highest abundant were the Agaricomycetes (18.4%), which represented the vast majority of Basidiomycota. The other highly abundant classes were all ascomycetous: Leotiomycetes (10.5%), Eurotiomycetes (8.4%), Dothideomycetes (8.2%) and Sordariomycetes (7.3%).

Archaeorhizomycetes SSU rRNAs were detected in 17 out of 28 metatranscriptomes and in 8 out of 11 soils (Fig. 1 and Table 1). The three sites where no Archaeorhizomycetes were detected were predominantly hypoxic, i.e. peatland and mofette soils. These metatranscriptomes had on average the lowest number of fungal reads, thus the detection limit could have been too high for Archaeorhizomycetes.

The overall relative abundance of Archaeorhizomycetes transcripts across all samples was 2.0% (Fig. 1). Exploring only metatranscriptomes containing Archaeorhizomycetes, this class represented on average 3.3% of SSU rRNAs. The maximum proportion recorded was 9.4%. Thus, these PCR-independent metatranscriptome results support the view that Archaeorhizomycetes are rather a low abundant fungal class in soils and that reports of their high abundance might be derived from a preferential amplification of their ITS or rRNA genes in PCR assays^13^. However, a particularly high abundance of Archaeorhizomycetes in PCR based studies was reported in alpine tundra^14^ and coniferous forest soils^3^, which were not included in our study. Furthermore, metatranscriptomics is not unbiased and shares some biases with DNA methods, such as protocol-dependent preferential nucleic acid extraction for certain taxonomic groups.

The relative abundance of Archaeorhizomycetes varied strongly between sites, with no SSU rRNA transcripts being detected in the predominantly hypoxic arctic peatland (PsS, PsK) and mofette (MO) soils (Fig. 1). Common to these soils was the low abundance of vascular plant roots, supporting once more the association of Archaeorhizomycetes with plant roots, although an obligate aerobic lifestyle could also result in such a distribution pattern. Remarkably, the highest relative abundance of Archaeorhizomycetes (average 5.6%) was found in soils of a former lead and zinc mining site (Fig. 1); with increasing heavy metal concentrations (MiL < MiM < MiH) having no effect on their relative abundance. These data indicate that Archaeorhizomycetes are tolerant to high concentrations of these metals, adding one additional piece of knowledge to their autecology. In the other surveyed sites, Archaeorhizomycetes abundance was minimal – up to ~1% for grassland (MR, RS, RB) and negligible <0.01% for beech forest (FL, FS) soils (Fig. 1).

Exploring seasonal patterns in the abundance of Archaeorhizomycetes SSU rRNAs, we observed a high relative abundance in samples collected in spring, which is in agreement with a study conducted in a Colorado alpine tundra that found a peak in abundance of Archaeorhizomycetes during spring^14^. Nevertheless, this conclusion is rather speculative as there was no seasonal sampling done for any of the soils surveyed in our study. To provide a well-founded description of the Archaeorhizomycetes seasonal variability more research is needed.

**Figure 2:**
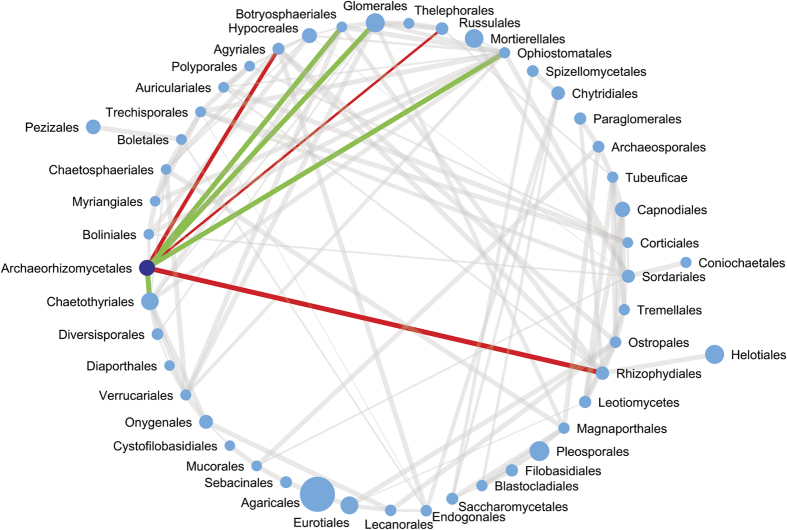
Co-occurrence network of significantly interacting fungal orders. Interactions with Archaeorhizomycetales are highlighted: positively (co-occurrence) interacting fungal orders are connected with green lines, negatively (mutual exclusion) interacting with red lines. The thickness of lines is proportional to significance of the interaction (q-value). The size of circle is proportional to the average relative abundance of fungal order in all datasets.

To identify possible interactions of Archaeorhizomycetes with other fungi we conducted network analysis (Fig. 2). We analysed the co-occurrence patterns of the 52 most abundant fungal orders in the metatranscriptomes using the CoNET algorithm^15^ in Cytoscape 3.0.2^16^ (for details see Methods section). Archaeorhizomycetes had a strong co-presence relationship with the arbuscular mycorrhizal Glomerales (Fig. 2; Table 2). However, we are not able to resolve whether both fungal groups interact directly (share of mycorrhizal niche or parasitism) or only share the affinity to root environment without explicit relationship. Also other positively interacting fungal orders have representatives with strong association to plants as either pathogens or endophytes (Table 2). The results of network analysis therefore further supported the hypothesis that Archaeorhizomycetes prefer to live in close vicinity of plant roots.

Co-occurrence analysis placed Archaeorhizomycetes among the top 10 potential keystone taxa (Table 3), thus Archaeorhizomycetes seem to play a non-negligible role in soil fungal communities despite their low relative abundance.

This study concludes Archaeorhizomycetes as fungal class with minor representation in transcriptionally active part of soil fungal communities in different terrestrial biomes. Yet, it highlights the need for more studies to elucidate more aspects of their ecology, as Archaeorhizomycetes were identified as putative key players in soil fungal communities.

## Methods

Metatranscriptome sequences were filtered using PRINSEQ^17^ discarding short and low quality sequences (<150 bp, min average quality <0.25; exception dataset RB: <80 bp, min average quality <0.25). Metatranscriptomes generated with Illumina HiSeq were subsampled to 1 million reads and screened for eukaryotic SSU rRNA transcripts using SortMeRNA^18^ with default settings and the Silva SSUref 111 database^19^. Metatranscriptomes generated with 454 pyrosequencing were not subsampled and not subjected to SortMeRNA. For fungal community profiling, whole metatranscriptome datasets (454 pyrosequencing) or eukaryotic SSU rRNA transcripts (Illumina) were subsequently compared with BLASTN against the Silvamod SSU rRNA reference database^20^ and taxonomically classified with CREST^20^ and MEGAN5^21^ using the lowest common ancestor algorithm (LCA parameters: minimum bit score 100, top percent 2). In addition, correctness of reads identified as belonging to the class Archaeorhizomycetes was manually verified with BLASTN searches of all identified reads against Archaeorhizomycetes SSU rRNA reference sequences in NCBI nt database (minimum query length 150 bp, minimum similarity with Archaeorhizomycetes reference sequences >92%; reference sequences: EU179934.1, EU179935.1, EU179933.1, EU179936.1, KF993708.1, JF836023.1, JF836020.1). The 92% cut off was chosen to be relaxed enough to allow acceptance of possible Archaeorhizomycetes transcripts slightly different to available Archaeorhizomycetes reference sequences, but on the other hand to avoid the false positive assignment of SSU rRNAs from related taxa to Archaeorhizomycetes.

The network analysis was done in Cytoscape 3.0.2^16^ with CoNET algorithm^15^. Only those fungal orders whose sum of sequences in all datasets was higher than 200 were included into the network analyses. The parameters and settings for network analyses in CoNET algorithm were: -parent_child_exclusion, -row_minocc 10, -correlations (Spearman, Pearson, mutual information, Bray-Curtis dissimilatory and Kullback-Leibler dissimilatory), threshold for edge selection was set to 1000 top and bottom. In randomization step, 100 iterations were calculated for edge scores. In following bootstrap step 100 iterations were calculated, unstable edges were filtered out, Brown method was chosen as P-value merging method and Benjaminihochberg method for multiple test correction. Resulting network for fungal community was visualized and analysed (i.e. degree of nodes, betweenness centrality, closeness centrality) in Cytoscape 3.0.2 and nodes tending to have high mean degree, low betweenness centrality, and high closeness centrality were identified as potential keystone orders^22^.

## Additional Information

**How to cite this article**: Choma, M. *et al*. Low abundance of Archaeorhizomycetes among fungi in soil metatranscriptomes. *Sci. Rep.* **6**, 38455; doi: 10.1038/srep38455 (2016).

**Publisher's note:** Springer Nature remains neutral with regard to jurisdictional claims in published maps and institutional affiliations.

## Figures and Tables

**Table 1:** General site and sampling description.

Site	Peatland soil “Knudsenheia”	Peatland soil “Solvatn”	Mofette	Mofette reference	Rothamsted grassland	Rotböhl	Forest Litter	Forest Soil	Mine L	Mine M	Mine H
Abbreviation	PsK	PsS	MO	MR	RS	RB	FL	FS	MiL	MiM	MiH
Location	Ny-Ålesund,	Ny-Ålesund,	Hartoušov,	Hartoušov,	Rothamsted,	Darmstadt,	Vienna woods,	Vienna woods,	Coto Txomin,	Coto Txomin,	Coto Txomin,
	Norway	Norway	Czech Republic	Czech Republic	United Kingdom	Germany	Austria	Austria	Spain	Spain	Spain
	(Svalbard)	(Svalbard)									
Climatic zone	Arctic	Arctic	Temperate	Temperate	Temperate	Temperate	Temperate	Temperate	Temperate	Temperate	Temperate
Biome	Fen wet land	Fen wet land	Floodplain	Floodplain	Grassland	Grassland	Temperate deciduous forest	Temperate deciduous forest	Shrubland	Shrubland	Shrubland
Dominant vegetation	Mosses	Mosses	*Filipendula ulmaria*	*Deschampsia cespitosa, Eriophorum vaginatum*	N.A.	N.A.	*Fagus sylvatica*	*Fagus sylvatica*	*Ulex europaeus*	*Festuca rubra*	*Festuca rubra*
Substrate type / Horizon	Organic peat (Top layer)	Organic peat (Top layer)	Organic soil	Gleic fluvisol	Mineral soil	Mineral soil	Litter horizon	Mineral soil (A horizon)	Mineral soil	Mineral soil	Mineral soil
pH	7.3	7.6	4.7	5.3	4.9	7.1	N.A.	4.5-5.1	3.9	5.6	5.9
Moisture (% soil dry weight)	1010	900	N.A	N.A.	33	32	18	43-64	52	49	30
# of replicates	2	2	3	3	2	1	2	4	3	3	3
Sampling time	August 2009	August 2009	July 2013	July 2013	July 2009	January 2006	May 2008	May 2008	March 2011	March 2011	March 2011
Sequencing method	454 GS FLX Titanium	454 GS FLX Titanium	Illumina HiSeq 2500	Illumina HiSeq 2500	454 GS FLX Titanium	454 GS 20	454 GS FLX	454 GS FLX	Illumina HiSeq 2000	Illumina HiSeq 2000	Illumina HiSeq 2000
Average eukaryotic SSU rRNA transcripts length	369	372	166	167	314	105	218	271	171	171	178
Average fungal SSU rRNA transcripts analysed	514	1632	1102	5086	2164	3287	28640	2187	4445	3014	2125
Average proportion of Archaeorhizomycetes SSU rRNA transcripts (%)	0.00	0.00	0.00	0.95	1.05	0.14	<0.01	<0.01	4.03	7.28	5.74
Reference	[9]	[9]	[12]	[12]	[10]	[7]	[10]	[10]	[11]	[11]	[11]

**Table 2:** Fungal orders significantly interacting with Archaeorhizomycetales, their assignment to phylum, prevailing lifestyle acc. to Tedersoo *et al*. 4, interaction q-value and mean relative abundance in all datasets.

Interacting order	Phylum	Prevailing lifestyle	q-value	Mean proportion (%)
***Co-presence***				
Botryosphaeriales	Ascomycota	Plant pathogens	1.2E-06	0.2
Chaetothyriales	Ascomycota	Saprotrophs, plant endophytes	6.4E-09	2.6
Glomerales	Glomeromycota	Arbuscular mycorrhiza	4.1E-07	3.1
Ophiostomatales	Ascomycota	Plant pathogens	7.4E-06	0.4
***Mutual exclusion***				
Agyriales	Ascomycota	Lichenicolous	1. E-02	0.6
Russulales	Basidiomycota	Ectomycorrhiza and saprotrophs	2.5E-02	0.9
Rhizophydiales	Chytridiomycota	Pathogens, saprotrophs	1.3E-06	1.2

**Table 3:** Potential keystone orders, their assignment to phyla, degree, betweenness centrality and closeness centrality.

Potential keystone order	Phylum	Degree	Betweenness centrality	Closeness centrality
Ophiostomatales	Ascomycota	12	0.13	0.45
Glomerales	Glomeromycota	9	0.12	0.42
Chaetothyriales	Ascomycota	9	0.05	0.41
Sordariales	Ascomycota	9	0.15	0.42
Trechisporales	Basidiomycota	8	0.11	0.42
Ostropales	Ascomycota	8	0.07	0.39
Rhizophydiales	Chytridiomycota	8	0.11	0.40
Botryosphaeriales	Ascomycota	7	0.03	0.41
Hypocreales	Ascomycota	7	0.03	0.41
Archaeorhizomycetales	Ascomycota	7	0.04	0.42

Orders are sorted according their degree (i.e. number of direct interactions with other fungal orders).

## References

[b1] Rosling A. . Archaeorhizomycetes: Unearthing an Ancient Class of Ubiquitous Soil Fungi. Science (80-.). 333, 876–879 (2011). 10.1126/science.1206958 21836015

[b2] Rosling A., Timling I. & Taylor D. L. In Genomics of Soil- and Plant-Associated Fungi (eds Horwitz B. A., Mukherjee P. K., Mukherjee M. & Kubicek C. P.) 36, 333–349 (Springer-Verlag Berlin Heidelberg, 2013).

[b3] Porter T. M. . Widespread occurrence and phylogenetic placement of a soil clone group adds a prominent new branch to the fungal tree of life. Mol. Phylogenet. Evol. 46, 635–644 (2008). 18032071 10.1016/j.ympev.2007.10.002

[b4] Tedersoo L. . Global diversity and geography of soil fungi. Science (80-.). 346, 1078 (2014). 10.1126/science.1256688 25430773

[b5] Schadt C. W. & Rosling A. Comment on ‘Global diversity and geography of soil fungi’. Science (80-.). 348, 1438–1438 (2015). 10.1126/science.aaa4269 26113712

[b6] Tedersoo L. . Response to Comment on ‘Global diversity and geography of soil fungi’. Science (80-.). 349, 936 (2015). 10.1126/science.aaa5594 26315429

[b7] Urich T. . Simultaneous assessment of soil microbial community structure and function through analysis of the meta-transcriptome. PLoS One 3 (2008). 10.1371/journal.pone.0002527 PMC2424134 18575584

[b8] Urich T. & Schleper C. In Handbook of Molecular Microbial Ecology I (ed de Brujin F.) 587–596 (John Wiley & Sons, Inc., 2011).

[b9] Tveit A., Schwacke R., Svenning M. M. & Urich T. Organic carbon transformations in high-Arctic peat soils: key functions and microorganisms. ISME J. 7, 299–311 (2013). 22955232 10.1038/ismej.2012.99 PMC3554415

[b10] Geisen S. . Metatranscriptomic census of active protists in soils. ISME J. doi: 10.1038/ismej.2015.30 (2015). PMC4579471 25822483

[b11] Epelde L., Lanzén A., Blanco F., Urich T. & Garbisu C. Adaptation of soil microbial community structure and function to chronic metal contamination at an abandoned Pb-Zn mine. FEMS Microbiol. Ecol. 91, 1–11 (2015). 10.1093/femsec/fiu007 25764532

[b12] Beulig F. . Altered carbon turnover processes and microbiomes in soils under long-term extremely high CO2 exposure. Nat. Microbiol. 1, 15025 (2016). 27571979 10.1038/nmicrobiol.2015.25

[b13] Tedersoo L. . Shotgun metagenomes and multiple primer pair-barcode combinations of amplicons reveal biases in metabarcoding analyses of fungi. MycoKeys 10, 1–43 (2015).

[b14] Schadt C. W., Martin A. P., Lipson D. a. & Schmidt S. K. Seasonal dynamics of previously unknown fungal lineages in tundra soils. Science 301, 1359–1361 (2003). 12958355 10.1126/science.1086940

[b15] Faust K. . Microbial co-occurrence relationships in the Human Microbiome. PLoS Comput. Biol. 8 (2012). 10.1371/journal.pcbi.1002606 PMC3395616 22807668

[b16] Shannon P. . Cytoscape: a software environment for integrated models of biomolecular interaction networks. Genome Res 13, 2498–2504 (2003). 14597658 10.1101/gr.1239303 PMC403769

[b17] Schmieder R. & Edwards R. Quality control and preprocessing of metagenomic datasets. Bioinformatics 27, 863–4 (2011). 21278185 10.1093/bioinformatics/btr026 PMC3051327

[b18] Kopylova E., Noé L. & Touzet H. SortMeRNA: fast and accurate filtering of ribosomal RNAs in metatranscriptomic data. Bioinformatics 28, 3211–7 (2012). 23071270 10.1093/bioinformatics/bts611

[b19] Quast C. . The SILVA ribosomal RNA gene database project: Improved data processing and web-based tools. Nucleic Acids Res. 41, 590–596 (2013). 10.1093/nar/gks1219 PMC3531112 23193283

[b20] Lanzén A. . CREST - Classification Resources for Environmental Sequence Tags. PLoS One 7 (2012). 10.1371/journal.pone.0049334 PMC3493522 23145153

[b21] Huson D. H., Auch A. F., Qi J. & Schuster S. C. MEGAN analysis of metagenomic data. Genome Res. 17, 377–86 (2007). 17255551 10.1101/gr.5969107 PMC1800929

[b22] Berry D. & Widder S. Deciphering microbial interactions and detecting keystone species with co-occurrence networks. Front. Microbiol. 5, 1–14 (2014). 24904535 10.3389/fmicb.2014.00219 PMC4033041

